# Design and Synthesis of Cu@CuS Yolk–Shell Structures with Enhanced Photocatalytic Activity

**DOI:** 10.1007/s40820-017-0135-7

**Published:** 2017-03-01

**Authors:** Qiuyan Li, Fan Wang, Linqiang Sun, Zhe Jiang, Tingting Ye, Meng Chen, Qiang Bai, Chao Wang, Xiguang Han

**Affiliations:** 1 Jiangsu Key Laboratory of Green Synthetic Chemistry for Functional Materials, Department of Chemistry, School of Chemistry and Chemical Engineering, Jiangsu Normal University, Xuzhou, 221116 People’s Republic of China; 2 College of Materials Science and Engineering, Qingdao University of Science and Technology, Qingdao, 266042 People’s Republic of China

**Keywords:** Nanomaterial, Yolk–shell structure, Cu@CuS, Photocatalysis

## Abstract

Non-spherical Cu@CuS yolk–shell structures are successfully obtained using Cu_2_O cube templates in a process combining rapid surface sulfidation followed by disproportionation of the Cu_2_O core upon treatment with a hydrochloric acid solution. By employing the above method, Cu@CuS yolk–shell structures with different morphologies, including octahedral, truncated octahedral, and cuboctahedral shapes, can be synthesized. The void space within the hollow structures provides a unique confined space, where the metallic copper present in the core of a shell can be protected from agglomeration and oxidation. Furthermore, the presence of metal copper in these hollow structures contributes to improvement in the photocatalytic properties of these materials. The application of these Cu@CuS structures indeed shows clearly improved photocatalytic performance.

**Electronic supplementary material:**

The online version of this article (doi:10.1007/s40820-017-0135-7) contains supplementary material, which is available to authorized users.

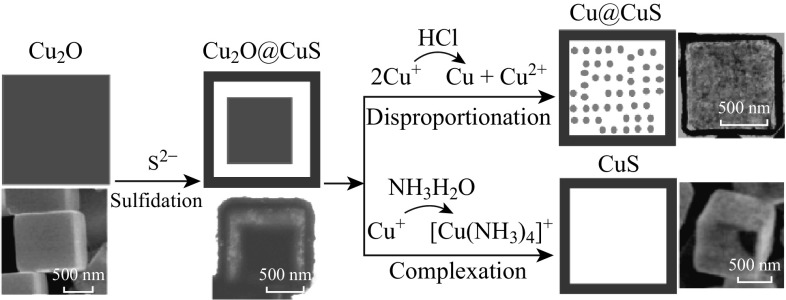

## Highlights

- Non-spherical Cu@CuS yolk–shell structures with different morphologies, including octahedral, truncated octahedral, and cuboctahedral shapes, are successfully obtained using a strategy combining shell sulfidation and core disproportionation reaction.
- The as-prepared Cu@CuS structures exhibited clearly remarkable photocatalytic performance.

## Introduction

Photocatalytic technology can be used to convert solar energy into chemical energy and constitutes a promising approach to combat both environmental pollution and the global energy shortage [1–4]. Metal sulfides have been drawing attention as new photocatalytic materials owing to their narrow band gap, good light adsorption properties, and excellent photocatalytic performance [5, 6]. In particular, copper sulfides (Cu_*x*_S with *x* = 1–2) are transition metal chalcogenides that have been found to be potentially useful materials for application in photocatalysis due to their low cost, high stability, and low cytotoxicity [7]. Recently, many varieties of copper sulfides micro-/nanostructures have been synthesized to improve their photocatalytic efficiency, including nanotubes [8], nanowires [9], nanorods [10], nanoplates [11], ball-flower [12], and hollow cages [13]. However, the photocatalytic activity of pure copper sulfides under visible light irradiation is not high enough for practical applications due to drawbacks associated with their narrow band gap, which results in the easy recombination of the photogenerated charges.

To address this issue, various methods have been developed, such as a controllable synthesis by doping with different ions [14, 15], sensitization by absorbed molecules or quantum dots [16, 17], and coupling with different band-gap semiconductors [18]. Among these, the modification of a photocatalyst with noble metals such as Pt, Au, and Ag is a meaningful and efficient strategy, since these metals can act as electron sinks to effectively transfer the photogenerated electrons, thus improving the corresponding photocatalytic performance [19–21]. Compared to noble metals, copper is particularly interesting due to its high conductivity combined with a much lower cost. However, deposition of copper on a photocatalyst surface is not always useful in photocatalysis since copper particles have a strong tendency to agglomerate and can be easily oxidized during the preparation and utilization processes [22].

Compared to other dense structures of the same size, yolk–shell nanostructures with a void space between the interior core and the mesoporous outer shell have attracted a great deal of attention due to their special structure and properties, such as low density, large surface area, and high loading capacity [23–28]. The void space within the yolk–shell structures provides a unique confined space, where a metal present as the yolk in the core of a shell can be protected from agglomeration and oxidation [29]. In general, various yolk–shell nanoparticles (NPs) have been synthesized by selectively scarifying the core/shell layer or converting the core materials via a disproportionation reaction [30], galvanic reaction [31, 32], or the Kirkendall effect [33]. However, most of the studies on the preparation of yolk–shell NPs focus on a noble metal, such as Au, Ag, and Pt, as the core and a metal oxide as the shell. Investigations on nanostructures using copper as the core and metal sulfides as the shell have been relatively limited. Therefore, it is still desirable to develop new efficient strategies to fabricate Cu@metal sulfides with high-performance photocatalytic activity.

Herein, we developed a strategy combining a shell sulfidation and core disproportionation reaction to synthesize uniform, non-spherical Cu@CuS yolk–shell structures. When evaluated as photocatalytic materials, the Cu@CuS structures showed a clear improvement in their photocatalytic performance.

## Experimental

### Chemicals

Copper(II) sulfate pentahydrate (CuSO_4_·5H_2_O), cupric acetate monohydrate [Cu(CH_3_COO)_2_·H_2_O], trisodium citrate dihydrate (Na_3_C_6_H_5_O_7_·2H_2_O), sodium hydroxide (NaOH), ascorbic acid (C_6_H_8_O_6_), sodium sulfide (Na_2_S·9H_2_O), hydrochloric acid (HCl), ammonia (NH_3_·H_2_O), and d-glucose (C_6_H_12_O_6_) were all purchased from Sinopharm Chemical Reagent Co., Ltd.

### Synthesis of the Materials

#### Synthesis of Cu_2_O Solid Cubes

In a typical synthesis, CuSO_4_·5H_2_O (0.375 g, 1.5 mmol) was dissolved in 100 mL of distilled water under vigorous stirring treatment to form solution 1. Subsequently, Na_3_C_6_H_5_O_7_·2H_2_O (0.15 g, 0.51 mmol) and NaOH (1 g, 25 mmol) were added to the above solution 1. Ascorbic acid (0.264 g 1.5 mmol) was dissolved in 50 mL of distilled water under ultrasonic treatment to form solution 2. Solution 2 was mixed with solution 1 at room temperature (25 °C), and the resulting mixture was stirred for 40 min at room temperature. A red product was obtained by centrifugation and washed with deionized water and ethanol. The product was dried in oven at 60 °C.

#### Preparation of Cu_2_O Solid Octahedrons

In a typical experiment, Cu(CH_3_COO)_2_·H_2_O (3 g, 15 mmol) was dissolved in deionized water (20 mL) under a constant stirring at 70 °C for 2 min. When a sodium hydroxide solution (10 mL, 9 M) was added to the above solution, a dark precipitate was produced. After stirring for 5 min, d-glucose powder (0.6 g) was then added with constant stirring at 70 °C for 60 min. A red product was obtained by centrifugation, washed with deionized water and ethanol, and then dried in oven at 60 °C. The preparation of Cu_2_O solid truncated octahedrons is similar to that of Cu_2_O solid octahedrons, except for the concentration and volume of the Cu(CH_3_COO)_2_·H_2_O (50 mL, 0.3 M) and sodium hydroxide (30 mL, 3 M) solutions. Cuboctahedral particles were also produced by a similar method, but the reaction time was reduced from 60 to 20 min.

#### Preparation of Cu_2_O@CuS Yolk–Shell Structures

In a typical synthesis, the Cu_2_O solid sample (70 mg) was added into distilled water to form solution 3, and then a sodium sulfide solution (1 mL, 0.6 M) was added to the above solution, and stirred for 5 min at room temperature. The black products were collected by centrifugation, washed with distilled water, and then dried at 60 °C for further characterization.

#### Preparation of Cu@CuS Yolk–Shell Structures

The above-synthesized black products (70 mg) were dissolved in 10 mL of distilled water under ultrasonic treatment to form solution 4. Hydrochloric acid (1 mL) was then added to solution 4 at 80 °C. After stirring for 2 h, the products were collected by centrifugation, washed, and dried at 60 °C for further characterization.

#### Preparation of CuS Hollow Structures

CuS particles were also prepared by a similar method together with Cu@CuS box structures, and ammonia was added to the black solution 4 instead of hydrochloric acid.

### Characterization

The composition and phase of the as-prepared products were acquired by powder X-ray diffraction (XRD) using a Panalytical X-pert diffractometer with CuKα radiation. The morphology and crystal structure of the as-prepared products were observed by scanning electron microscopy (SEM, SU8010) and high-resolution transmission electron microscopy (HRTEM, JEM-2100) with an acceleration voltage of 200 kV. All TEM samples were prepared by depositing a drop of the diluted suspensions in ethanol on a carbon-film-coated copper grid.

### Photocatalysis

The photodegradation efficiency of methylene blue (MB) in aqueous solution was measured under sunlight irradiation (300 W xenon lamp). All experiments were carried out at the temperature of 25 ± 2 °C. Typically, 15 mg of Cu@CuS yolk–shell sample was dispersed in 50 mL of MB aqueous solution (0.02 M) under ultrasonic irradiation to form a suspension, which was magnetically stirred for 30 min in the dark. At regular irradiation time intervals, the dispersion was sampled and centrifuged to separate the residue. The photodegradation efficiency was monitored by measuring the absorbance of the centrifuged solutions at the maximum absorption wavelength of 664 nm using UV–Vis spectroscopy (SHIMADZU, UV-2100) at room temperature.

## Results and Discussion

**Fig. 1:**
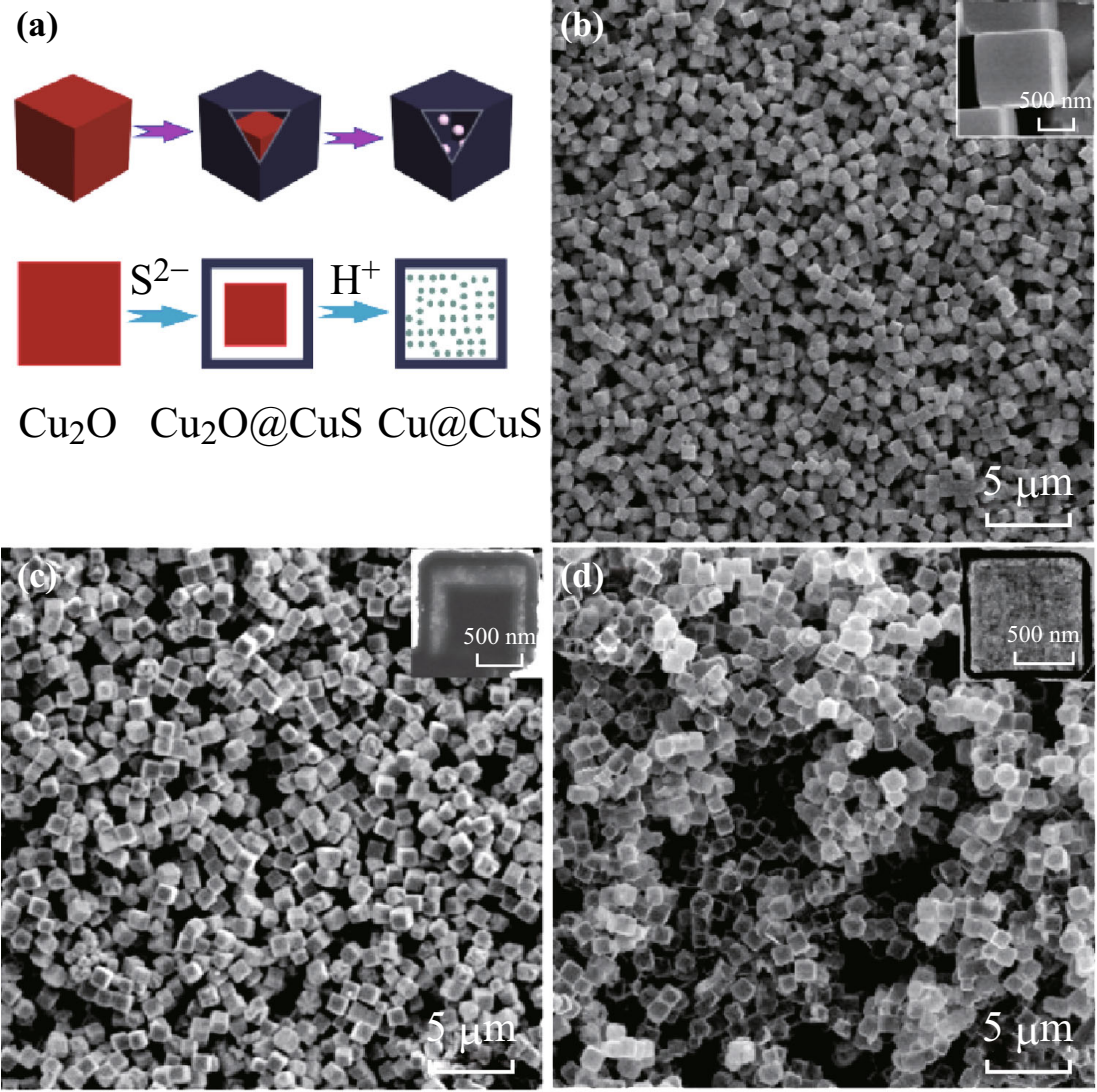
**a** Schematic illustration of the formation mechanism of cubic Cu@CuS yolk–shell structures. SEM images of **b** Cu_2_O *solid cubes* (the *inset* shows the corresponding enlarged image), **c** Cu_2_O@CuS yolk–shell structures, and **d** single-shell Cu@CuS yolk–shell structures. The *insets* in **c** and **d** show the corresponding TEM images

**Fig. 2:**
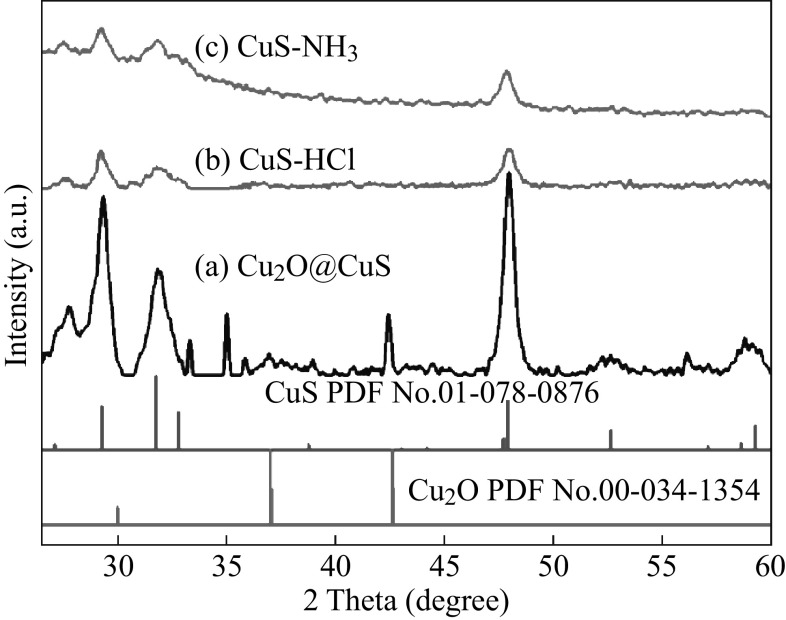
XRD patterns of **a** cubic Cu_2_O@CuS, **b** products after hydrochloric acid reaction, and **c** products after ammonia reaction

Our strategy for the formation of Cu@CuS yolk–shell structures is shown in Fig. 1a. In the first step, the Cu_2_O cubes used as precursors were prepared by a facile solvothermal method. The XRD pattern (Fig. S1) of the obtained products corresponds to the cubic Cu_2_O phase (JCPDS card no. 00-034-1354, α = 0.4217 nm), indicating that Cu_2_O was successfully synthesized. As shown in Fig. 1b, the synthesized uniform Cu_2_O cubes exhibit a smooth surface and have a mean size of about 1 μm. The Cu_2_O cubes can be easily converted into Cu_2_O@CuS yolk–shell structures by a simple surface sulfidation process using Na_2_S as sulfur source. XRD analysis was used to elucidate the composition and crystal phase of the as-prepared products. As shown in Fig. 2a, most of the diffraction peaks can be ascribed to hexagonal CuS (JCPDS card no. 01-078-0876), while a part of the peaks (marked with a star) corresponds to the primitive cubic Cu_2_O (JCPDS card no. 00-034-1354). Therefore, the as-prepared products are composite of CuS and Cu_2_O phases, and the reaction equation can be described as: 2Cu_2_O + 4S^2−^ + O_2_ + 4H_2_O → 4CuS + 8OH^−^ [34]. The morphologies of the products were analyzed by FESEM and TEM. As shown in Fig. 1c, the morphologies of the products still retain uniform cubes after the sulfidation process. The TEM images (inset of Fig. 1c) confirm the formation of yolk–shell structures by the obvious contrast between the cavity and the shell, which has a thickness of approximately 50 nm. The formation process of the yolk–shell structures can be divided into a sulfidation step and the Kirkendall effect. Firstly, the sulfide ions released from Na_2_S in the aqueous solution react with the metal ions on the surface of the Cu_2_O cubes to form a thin layer of Cu sulfides, and then the outward diffused metal ions become dominant compared to the inward diffusion of S^2−^ based on the Kirkendall effect, leading to the formation of a well-defined gap between the shell and the Cu_2_O core. Cuprous compounds can go through a disproportionation reaction to form Cu metal and Cu^2+^ ions under acidic conditions. Therefore, the cubic Cu_2_O core encapsulated in this system can be used to synthesize spatially confined Cu particles by a disproportionation reaction. When the Cu_2_O@CuS particles were added to a hydrochloric acid solution, the color of the solution turned blue-green (Fig. S2), indicating that the Cu^2+^ ions generated from the disproportionation reaction can diffuse to the solution through the CuS shell. The SEM and TEM images (Fig. 1d) reveal that the Cu_2_O core disappeared and the corresponding hollow cubes could be generated after acidic treatment, suggesting the conversion of Cu_2_O cubes to Cu crystals. However, no diffraction peaks corresponding to Cu were found in the XRD pattern (Fig. 2b), most likely due to its low content and poor crystalline. Actually, the Cu_2_O core of the Cu_2_O@CuS particles could be completely dissolved by ammonia complexation reaction to form [Cu(NH_3_)_4_]^+^ and hollow CuS boxes. The composition and morphology of the products were examined by XRD (Fig. 2c), SEM, and TEM analyses (Fig. S3).

**Fig. 3:**
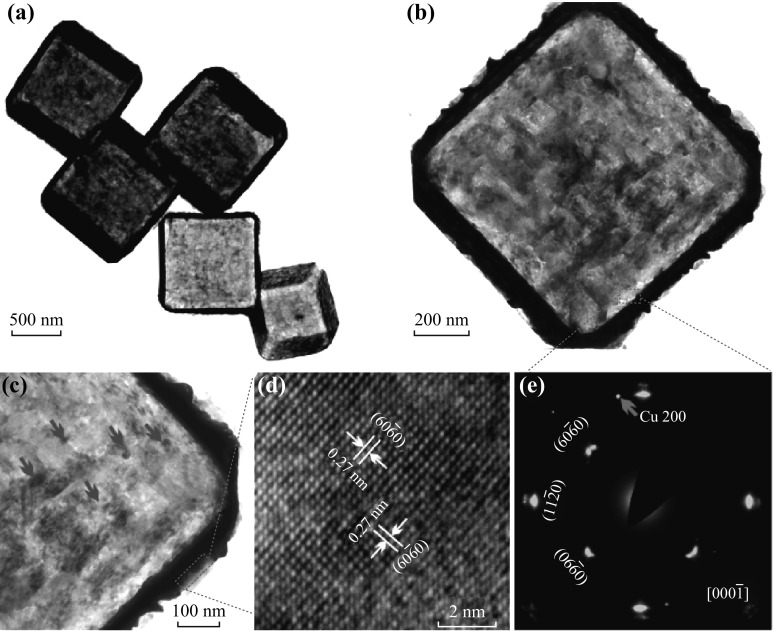
**a** Typical TEM image of cubic CuS@Cu yolk–shell structures. **b**, **c** Different magnification of the TEM image of individual Cu@CuS yolk–shell structures. **d** HRTEM image taken from the shell of a Cu@CuS yolk–shell structure. **e** The corresponding SAED pattern

In order to further confirm the composition and structural information of the Cu@CuS structures, the products were characterized by HRTEM and selected-area electron diffraction (SAED). In agreement with the FESEM findings, a high uniformity of the hollow cubes can be seen in the TEM images (Fig. 3a). Moreover, the inner cavities are clearly revealed by the sharp contrast between shells and hollow interiors. The TEM images at higher magnifications depict the inner cavities of the hollow cubes (Fig. 3b). Interestingly, it appears that some small particles with a size of several nanometers are present in the cavities (Fig. 3c). The SAED pattern corresponding to the square shell is displayed in Fig. 3e, and it shows that the square shell has a single crystalline characteristic, and it can be indexed to a primitive hexagonal phase of CuS viewed along the $[000\overline{1} ]$ zone axis. Moreover, some additional spots correspond well to the (200) reflections of the face-centered cubic Cu. The detailed microstructure of the square shell was further investigated by HRTEM. As shown in Fig. 3d, the corresponding lattice spacing is 0.27 nm, which is in good agreement with the *d* value of the $(60\overline{6} 0)$ facets of the hexagonal phase of CuS. The electron diffraction and HRTEM results further confirm that the as-prepared products are comprised of CuS and Cu.

**Fig. 4:**
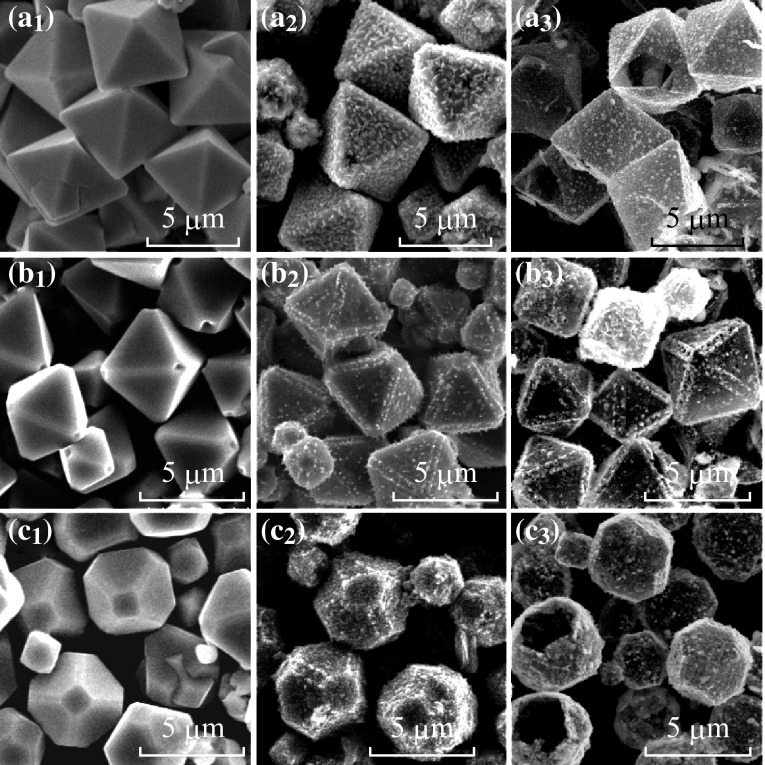
SEM images of different morphologies of Cu_2_O before and after the sulfidation–disproportionation process: **a** octahedral shape, **b** truncated octahedral shape, and **c** cuboctahedral shape

**Fig. 5:**
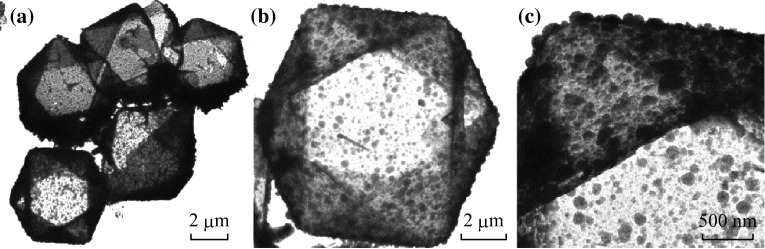
TEM images of Cu@CuS octahedrons at different magnifications

This shell sulfidation and core disproportionation strategy can also be used to synthesize Cu@CuS yolk–shell structures with different morphologies (octahedral, truncated octahedral, and cuboctahedral). Figure 4 shows the SEM images of the different morphologies of Cu_2_O before and after the sulfidation and disproportionation processes. Firstly, different shapes of the Cu_2_O particles with a smooth surface were synthesized by changing the reaction conditions (Fig. 4a_1_–c_1_). After the sulfidation treatment, the surface of the particles became rough (Fig. 4a_2_–c_2_). Selecting the octahedral shape as example XRD analysis (Fig. S4) was carried out to determine the structure and composition of the obtained samples. The diffraction peaks can be ascribed to hexagonal CuS and cubic Cu_2_O, indicating that the as-prepared products are composites of CuS and Cu_2_O phases. According to the above strategy, Cu_2_O can be used to synthesize spatially confined Cu particles by a disproportionation reaction. From the SEM images (Fig. 4a_3_–c_3_), the products still retain the original morphologies and the interior cavity is formed after the disproportionation reaction. The TEM images (Fig. 5a–c) of Cu_2_O octahedrons depict the inner cavities, and some small Cu particles appear in the cavities. The color of the solution turned blue-green (Fig. S5), which indicates that the Cu_2_O in the core has disappeared. Therefore, Cu@CuS yolk–shell structures with different morphologies can be synthesized utilizing this disproportionation method (Fig. 4b, c).

**Fig. 6:**
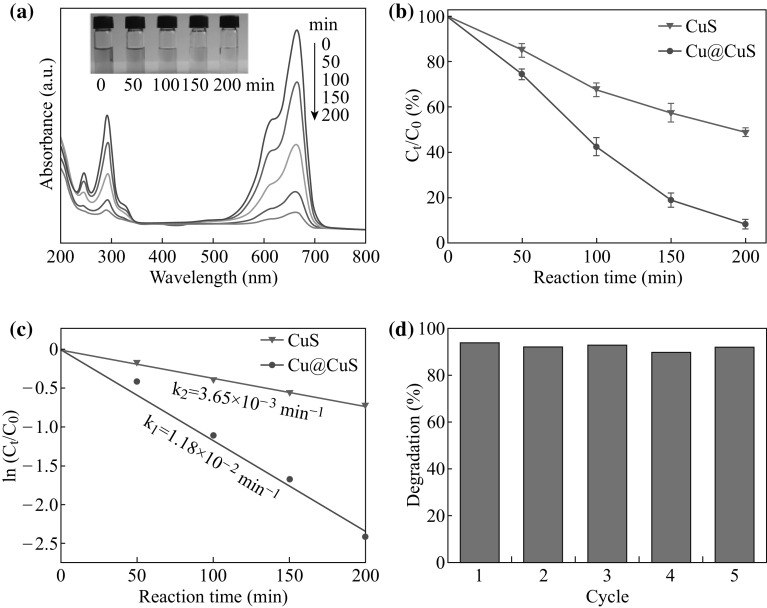
**a** UV–Vis absorption spectra of MB as a function of the xenon lamp (simulated sunlight) irradiation time for Cu@Cu_2_O cubes. The *inset* shows the corresponding photographs of MB irradiated by a xenon lamp for different periods of time. **b** Photocatalytic degradation curves of MB over different photocatalysts. **c** Plot of ln(*C*_t_/*C*_0_) as a function of time over different catalysts. **d** Cycle stability of Cu@Cu_2_O cubes

The photocatalytic activity of the Cu@CuS cubes was evaluated by MB degradation under sunlight irradiation. As shown in Fig. 6a, when the Cu@CuS cubes were used as photocatalyst, the degradation could be finished within 200 min, and the photographs indicate that the solution became colorless after 200 min (inset Fig. 6a). The normalized temporal concentration changes (*C*_t_/*C*_0_) of MB during the photocatalytic process are proportional to the normalized maximum absorbance (*A*_t_/*A*_0_), which can be derived from the change in the MB absorption profile at a given time interval. Figure 4b clearly shows that the photocatalytic activity of the Cu@CuS cubes is higher than that of the CuS cubes. A linear relation of ln(*C*_t_/*C*_0_) versus reaction time is observed, implying that the photocatalytic reaction can be considered as a pseudo-first-order reaction (Fig. 4c). The cubic Cu@CuS yolk–shell catalysts showed the fastest reaction rate, and the apparent kinetic rate constant was estimated to be 1.18 × 10^−2^ min^−1^, which is higher than that of the CuS cubes (3.65 × 10^−3^ min^−1^). In order to evaluate the catalytic stability, we carried out the cycle performance of double-shell structure Cu@CuS cubic catalysts for the degradation of MB under solar light irradiation (Fig. 4d). The results reveal that the Cu@CuS cubic structures possess an outstanding catalytic stability, which remains above 90% in the photodegradation of MB after five cycle tests.

**Fig. 7:**
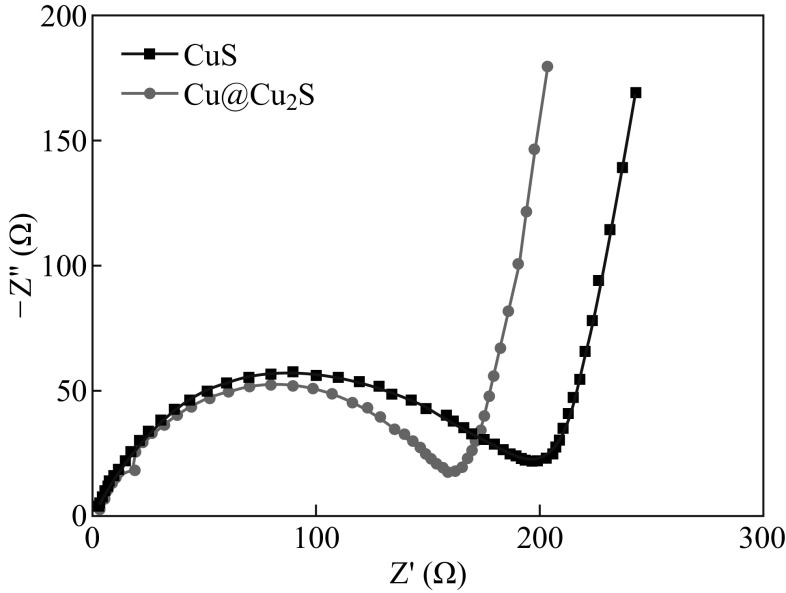
Nyquist plots of CuS and Cu@CuS cube samples

The enhanced photocatalytic activity of Cu@CuS cubes might be mainly ascribed to a reduced electron–hole pair recombination owing to the Cu/CuS hybrid nanostructures, as confirmed by EIS measurements (Fig. 7). The low charge-transfer resistance (*R*_ct_) indicates that the coupled system of semiconductors decreases the layer resistance in the solid-state interface and the charge-transfer resistance on the surface. Therefore, the above results indicate that the present copper can act as an electron sink to effectively transfer the photogenerated electrons of the photocatalyst (Cu_2_S), which may slow down the recombination of photogenerated electrons and holes to provide a high photocatalytic activity [35, 36].

## Conclusions

In summary, we have developed a new and efficient shell sulfidation and core disproportionation strategy for the synthesis of non-spherical Cu@CuS yolk–shell structures. This method can be used to fabricate Cu@CuS yolk–shell structures with different morphologies (cubic, octahedral, and cuboctahedral). The void space in the hollow structures provides a unique confined space, where a metal copper present in the core of a shell can be protected from agglomeration and oxidation. Furthermore, due to the presence of metal copper and hollow structures, the Cu@CuS yolk–shell structures manifest excellent photocatalytic activity and stability under sunlight irradiation.

## Electronic supplementary material

Below is the link to the electronic supplementary material.

Supplementary material 1 (PDF 538 kb)
