# Supplementary material for: Design and Synthesis of Cu@CuS Yolk–Shell Structures with Enhanced Photocatalytic Activity
Source: Nanomicro Lett. 2017 Mar 1;9(3):35. doi: 10.1007/s40820-017-0135-7 (PMC6199031; doi:10.1007/s40820-017-0135-7)
Supplement: Supplementary file 1 — Supplementary material 1 (PDF 538 kb) [file 40820_2017_135_MOESM1_ESM.pdf]

**Supplementary Information for**

# Design and Synthesis of Cu@CuS Yolk-Shell Structures with Enhanced Photocatalytic Activity

Qiuyan Li[1, #], Fan Wang[1, #], Linqiang Sun[1], Zhe Jiang[1], Tingting Ye[1], Meng Chen[1], Qiang Bai[2], Chao Wang[1], Xiguang Han[1, *]

[1]Jiangsu Key Laboratory of Green Synthetic Chemistry for Functional Materials, Department of Chemistry, School of Chemistry and Chemical Engineering, Jiangsu Normal University, Xuzhou 221116, People's Republic of China

[2]College of Materials Science and Engineering, Qingdao University of Science and Technology, Qingdao 266042, People's Republic of China

E-mail: xghan@jsnu.edu.cn

[#]These authors contributed equally.

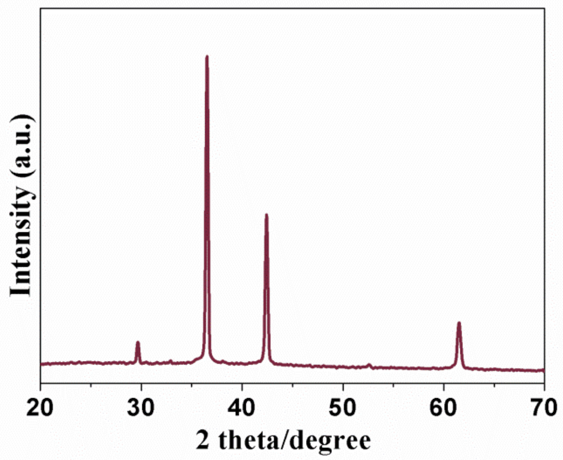


**Fig. S1** XRD pattern of the as-prepared $Cu_2O$ cubes

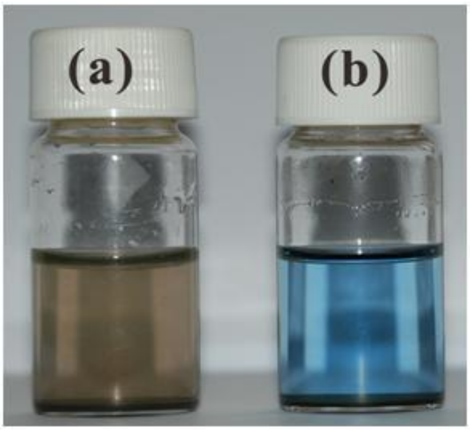


**Fig. S2** Photographs of $Cu_2O$@CuS cubes solution **a** before and **b** after disproportionation reaction

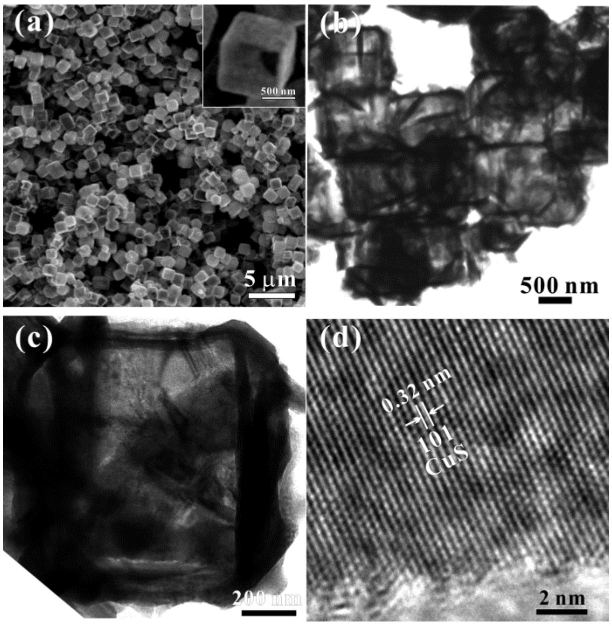


**Fig. S3 a** SEM image CuS hollow boxes synthesized by ammonia complexation reaction. **b, c** The corresponding TEM images. **d** HRTEM image

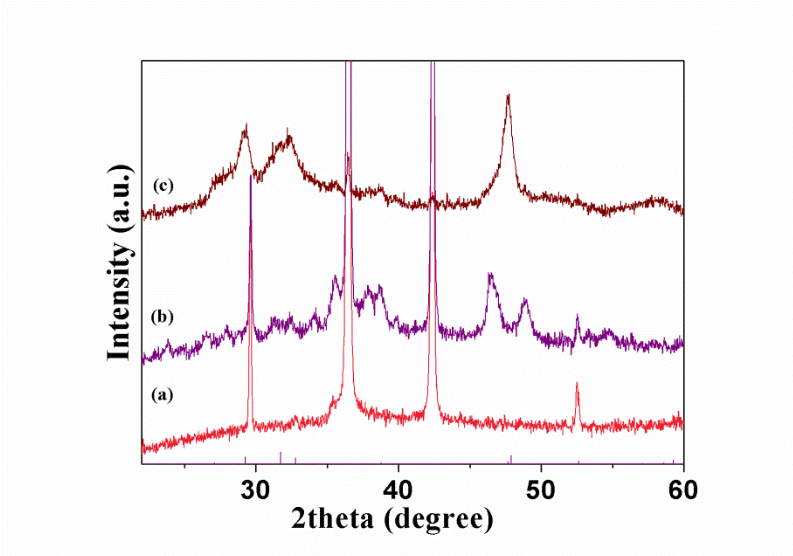


**Fig. S4** XRD patterns of **a** octahedral $Cu_2O$, **b** octahedral $Cu_2O$@CuS, and **c** octahedral Cu@CuS after hydrochloric acid reaction

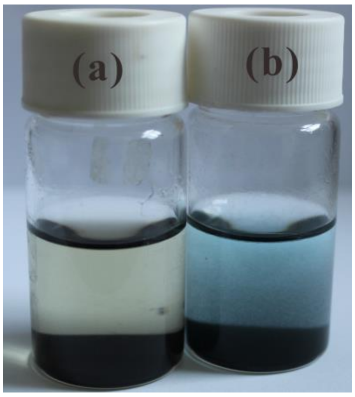


**Fig. S5** Photographs of $Cu_2O$@CuS octahedron solution **a** before and **b** after disproportionation reaction
